# ﻿A review of the spider genus *Sinoalaria* (Araneae, Theridiosomatidae), with the descriptions of four new species and two new combinations

**DOI:** 10.3897/zookeys.1173.105123

**Published:** 2023-08-07

**Authors:** Jianshuang Zhang, Chengcheng Feng, Hao Yu, Yucheng Lin

**Affiliations:** 1 School of Life Sciences, Guizhou Normal University, Guiyang, China; 2 Key Laboratory of Bio-resources and Eco-environment (Ministry of Education), College of Life Sciences, Sichuan University, Chengdu, China; 3 The Sichuan Key Laboratory for Conservation Biology of Endangered Wildlife, Sichuan University, Chengdu, China

**Keywords:** Araneoidea, identification key, Southeast Asia, southern China, morphology

## Abstract

The spider genus *Sinoalaria* Zhao & Li, 2014 is redefined and reviewed. A total of ten species are studied, including four new species: *S.chi* Yu & Lin, **sp. nov.** (♂♀), *S.shenhei* Yu & Lin, **sp. nov.** (♀), *S.shuidi* Yu & Lin, **sp. nov.** (♀), *S.xiaotu* Yu & Lin, **sp. nov.** (♂♀). Two new combinations are proposed: *Sinoalarianitida* (Zhao & Li, 2012), **comb. nov.** and *S.prolata* (Zhao & Li, 2012), **comb. nov.**, both transferred from *Karstia* Chen, 2010. The material of six known species were re-examined and photographed, including the type species, *S.chengguanensis* (Zhao & Li, 2012). A key is provided for all species of the genus, as well as diagnoses, illustrations, and a distribution map.

## ﻿Introduction

*Sinoalaria* Zhao & Li, 2014 is a replacement name for *Alaria* Zhao & Li, 2012, which was originally erected as a monotypic genus based on *A.chengguanensis* Zhao & Li, 2012 from China. It is a relatively small genus, distributed exclusively in South East Asia, with only four known species described so far (WSC 2023). All but the type species are from Laos and were described by Lin et al. (2014): *S.bicornis* (Lin, Li & Jäger, 2014), *S.cavernicola* (Lin, Li & Jäger, 2014) and *S.navicularis* (Lin, Li & Jäger, 2014). However, after 2014, no new species of this genus have been reported worldwide, and the diversity of this genus is still insufficiently known.

This paper reports our findings on the study of recently available samples from southwest China, Vietnam, Laos, and Thailand, which revealed four new species of *Sinoalaria*. Furthermore, two combinations are proposed based on morphological evidence and a preliminary molecular phylogeny (unpublished): *S.nitida* (Zhao & Li, 2012), comb. nov. and *S.prolata* (Zhao & Li, 2012), comb. nov., ex *Karstia* Chen, 2010. With that, the total number of *Sinoalaria* species reaches ten species. The goals of this paper are: 1) to conduct a comprehensive review of the genus *Sinoalaria*, provide an identification key and a distribution map for all species; 2) to describe the four new species under the name of *S.chi* Yu & Lin, sp. nov., *S.shenhei* Yu & Lin, sp. nov., *S.shuidi* Yu & Lin, sp. nov. and *S.xiaotu* Yu & Lin, sp. nov.; 3) to re-illustrate all six known species, including the type species and the two combinations.

## ﻿Materials and methods

All specimens were preserved in 95% ethanol. Specimens were examined and measured with a Leica M205 C stereomicroscope. Further details were studied with an Olympus BX43 compound microscope. Male and female genitalia were examined and illustrated after dissection. Epigynes were removed and cleared in lactic acid before illustration. All vulvae were imaged after being embedded in Arabic gum. Photos were taken with a Canon EOS 60D wide zoom digital camera (8.5 megapixels) mounted on an Olympus BX43 stereomicroscope. The images were montaged using Helicon Focus 3.10 (Khmelik et al. 2006) image stacking software. All measurements in the paper are in millimeters. Leg measurements are given in the following sequence: total length (femur, patella, tibia, metatarsus, and tarsus).

The distribution map was generated with ArcGIS 10.5 (Environmental Systems Research Institute, Inc.). Locality coordinates for all species are copied from the original publications (see Zhao and Li 2012; Lin et al. 2014).

All examined materials are deposited in the Natural History Museum of Sichuan University in Chengdu (**NHMSU**), China, expect for the types of *Sinoalariachengguanensis* and *S.prolata* in the Institute of Zoology, Chinese Academy of Sciences, Beijing, China (**IZCAS**).

## ﻿Taxonomy

**Family Theridiosomatidae Simon, 1881**

### ﻿Genus Sinoalaria

Taxon classificationAnimaliaAraneaeTheridiosomatidae

Zhao & Li, 2014

F7FC6695-6793-515D-917A-7C49C7247FC7

Alaria Zhao & Li, 2012: 7 (type species Alariachengguanensis Zhao & Li, 2012, by original designation).
Sinoalaria Zhao & Li, 2014: 41 (replacement name for Alaria Zhao & Li, 2012, preoccupied in Platyhelminthes by Schrank 1788).

#### Diagnosis.

**Figure 1.**
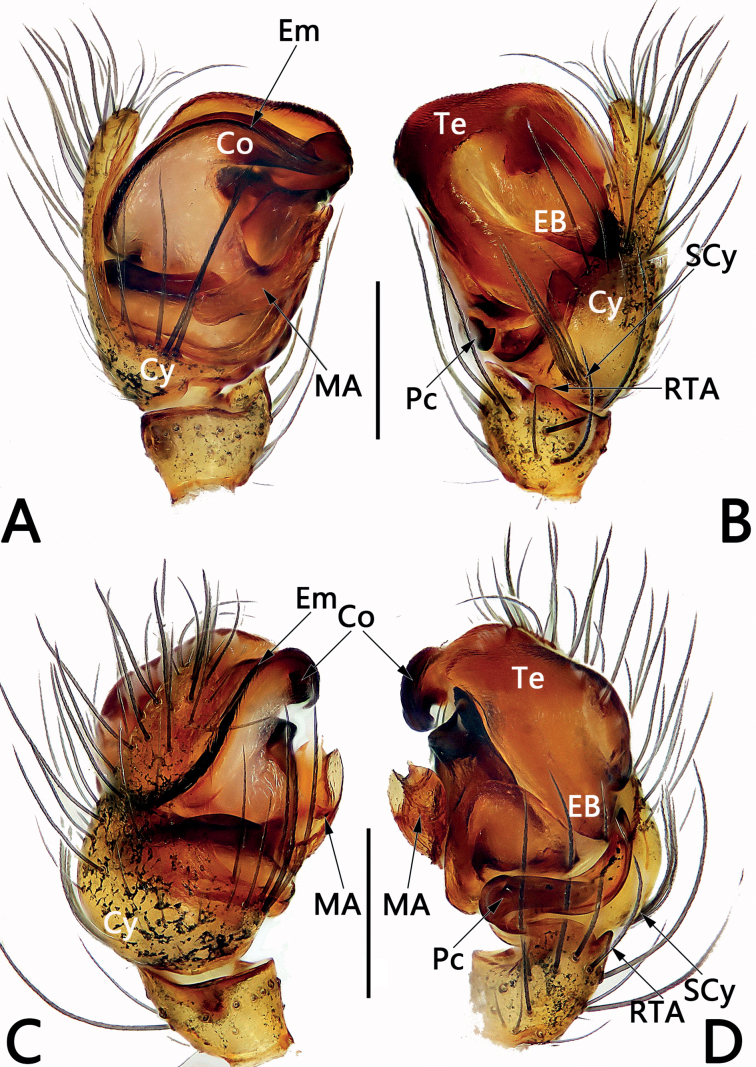
Male palp of *Sinoalariabicornis* (Lin, Li & Jäger, 2014) **A** prolateral view **B** retrolateral view **C** dorsal view **D** ventral view. Abbreviations: Co = conductor; Cy = cymbium; EB = embolic base; Em = embolus; MA = median apophysis; Pc = paracymbium; RTA = retrolateral tibial apophysis; SCy = setae on cymbium; Te = tegulum. Scale bars: 0.20 mm.

**Figure 2.**
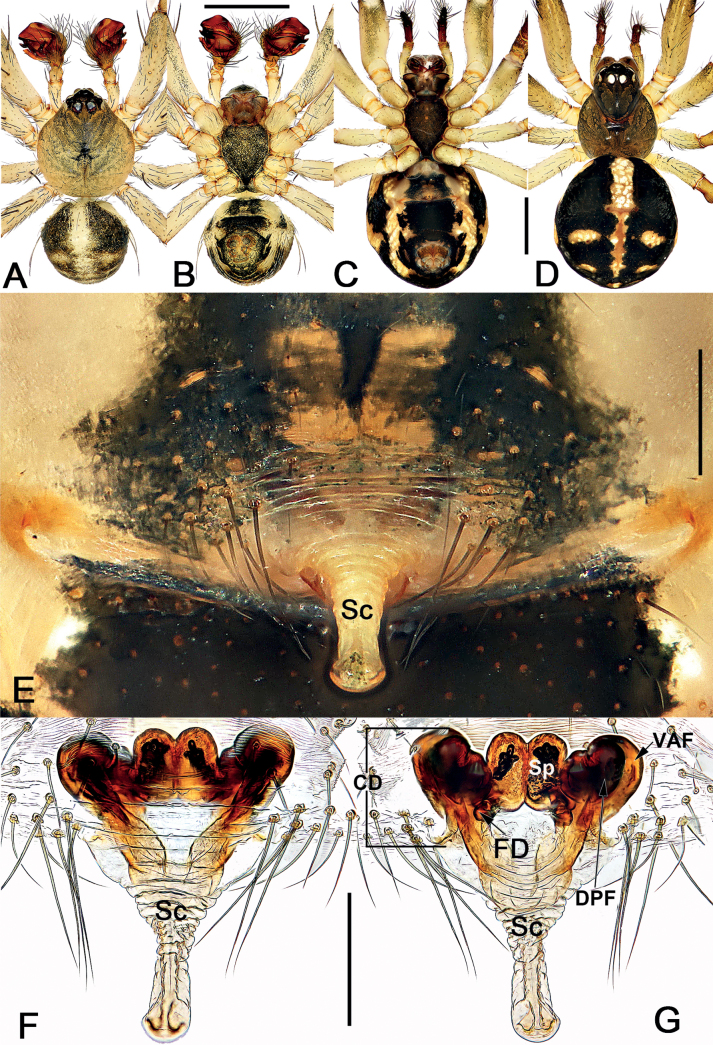
*Sinoalariabicornis* (Lin, Li & Jäger, 2014), male habitus (**A, B**), female habitus (**C, D**) and epigyne (**E–G**) **A** dorsal view **B** ventral view **C** ventral view **D** dorsal view **E** intact, ventral view **F** cleared, ventral view **G** cleared, dorsal view. Abbreviations: CD = copulatory duct; DPF = dorsal and posterior fold of copulatory duct; FD = fertilization duct; Sc = scape; Sp = spermatheca; VAF = ventral and anterior fold of copulatory duct. Scale bars: 0.50 mm (**A–D**); 0.20 mm (**E–G**).

**Figure 3.**
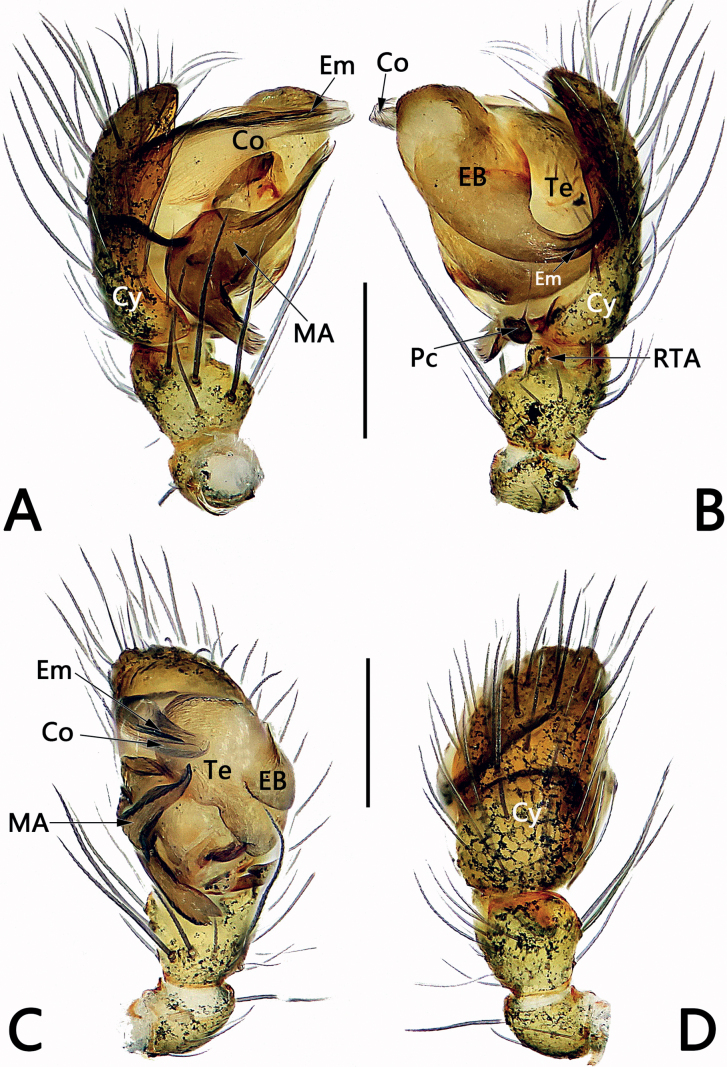
Male palp of *Sinoalariacavernicola* (Lin, Li & Jäger, 2014) **A** prolateral view **B** retrolateral view **C** ventral view **D** dorsal view. Abbreviations: Co = conductor; Cy = cymbium; EB = embolic base; Em = embolus; MA = median apophysis; Pc = paracymbium; RTA = retrolateral tibial apophysis; Te = tegulum. Scale bars: 0.20 mm.

**Figure 4.**
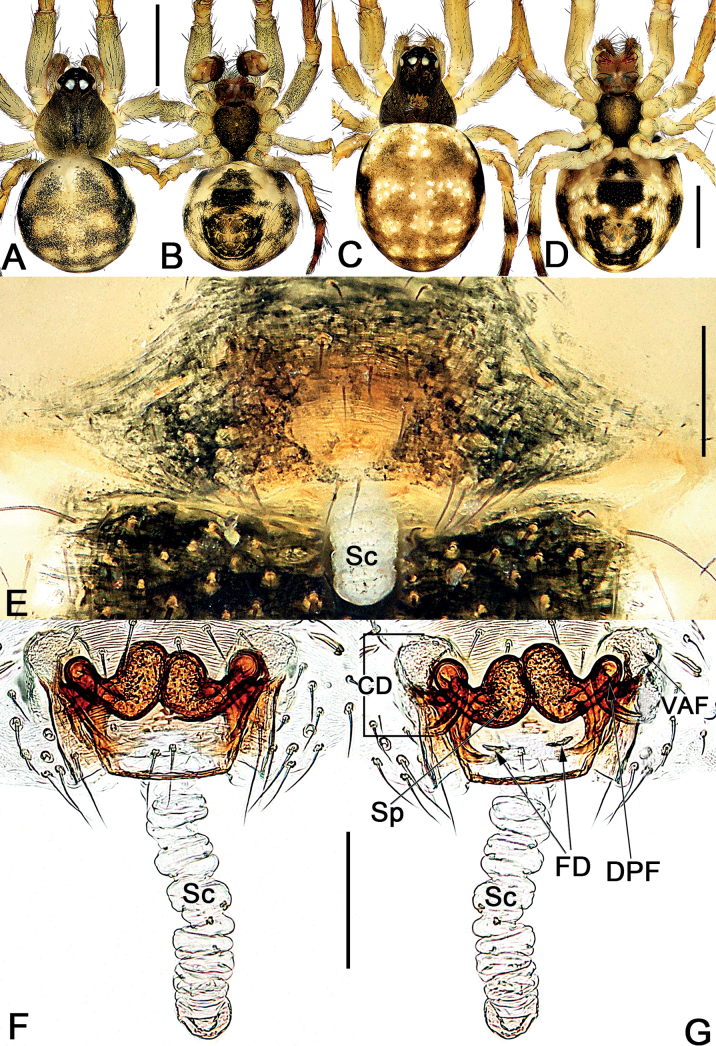
*Sinoalariacavernicola* (Lin, Li & Jäger, 2014), male habitus (**A, B**), female habitus (**C, D**) and epigyne (**E–G**) **A** dorsal view **B** ventral view **C** dorsal view **D** ventral view **E** intact, ventral view **F** cleared, ventral view **G** cleared, dorsal view. Abbreviations: CD = copulatory duct; DPF = dorsal and posterior fold of copulatory duct; FD = fertilization duct; Sc = scape; Sp = spermatheca; VAF = ventral and anterior fold of copulatory duct. Scale bars: 0.50 mm (**A–D**); 0.20 mm (**E–G**).

**Figure 5.**
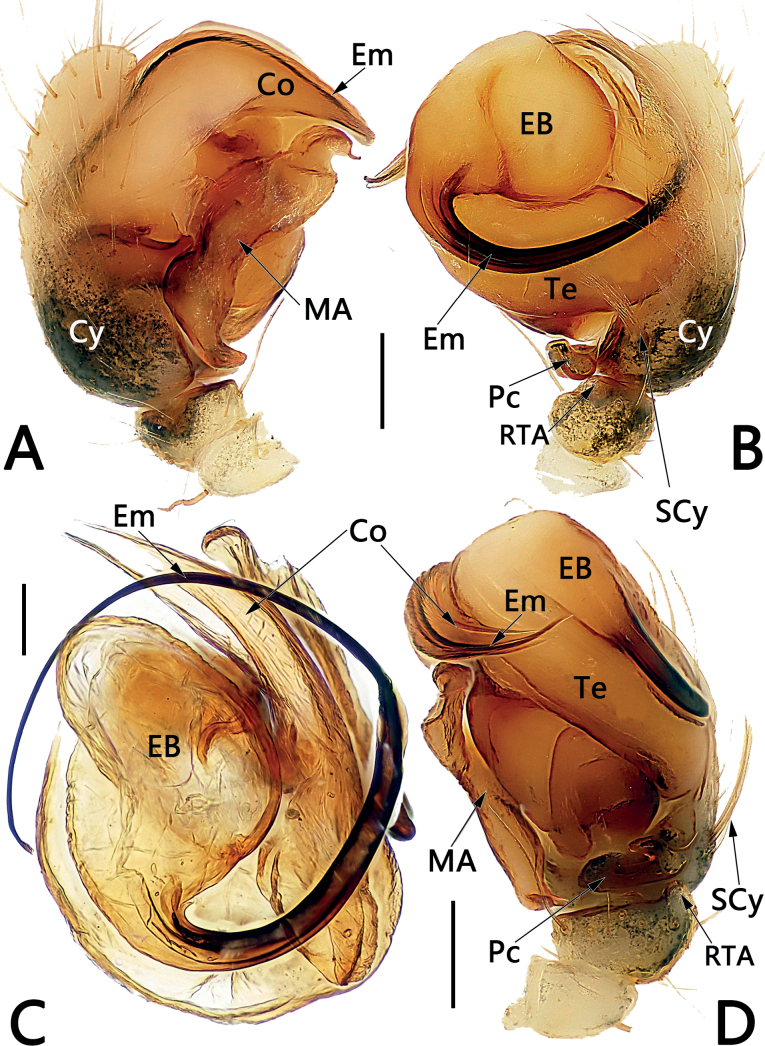
Male palp of *Sinoalariachengguanensis* (Zhao & Li, 2012) **A** prolateral view **B** retrolateral view **C** embolic division, dorsal view **D** ventral view. Abbreviations: Co = conductor; Cy = cymbium; EB = embolic base; Em = embolus; MA = median apophysis; Pc = paracymbium; RTA = retrolateral tibial apophysis; SCy = setae on cymbium; Te = tegulum. Scale bars: 0.20 mm.

**Figure 6.**
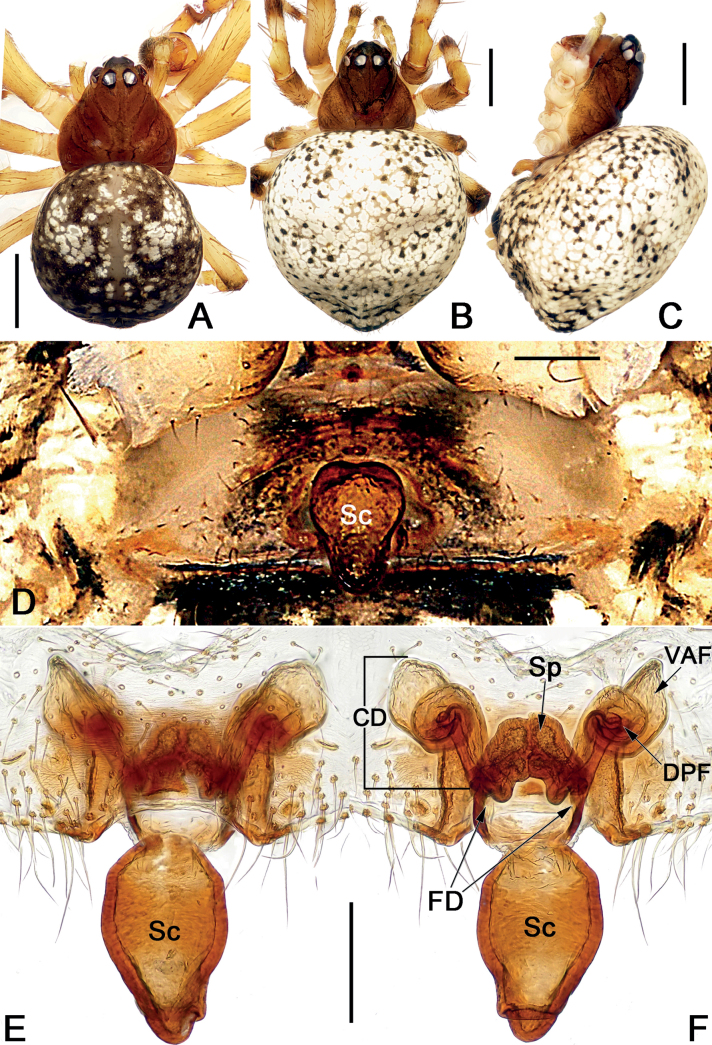
*Sinoalariachengguanensis* (Zhao & Li, 2012), male habitus (**A**), female habitus (**B, C**) and epigyne (**D–F**) **A** dorsal view **B** dorsal view **C** lateral view **D** intact, ventral view **E** cleared, ventral view **F** cleared, dorsal view. Abbreviations: CD = copulatory duct; DPF = dorsal and posterior fold of copulatory duct; FD = fertilization duct; Sc = scape; Sp = spermatheca; VAF = ventral and anterior fold of copulatory duct. Scale bars: 0.50 mm (**A–C**); 0.20 mm (**D–F**).

**Figure 7.**
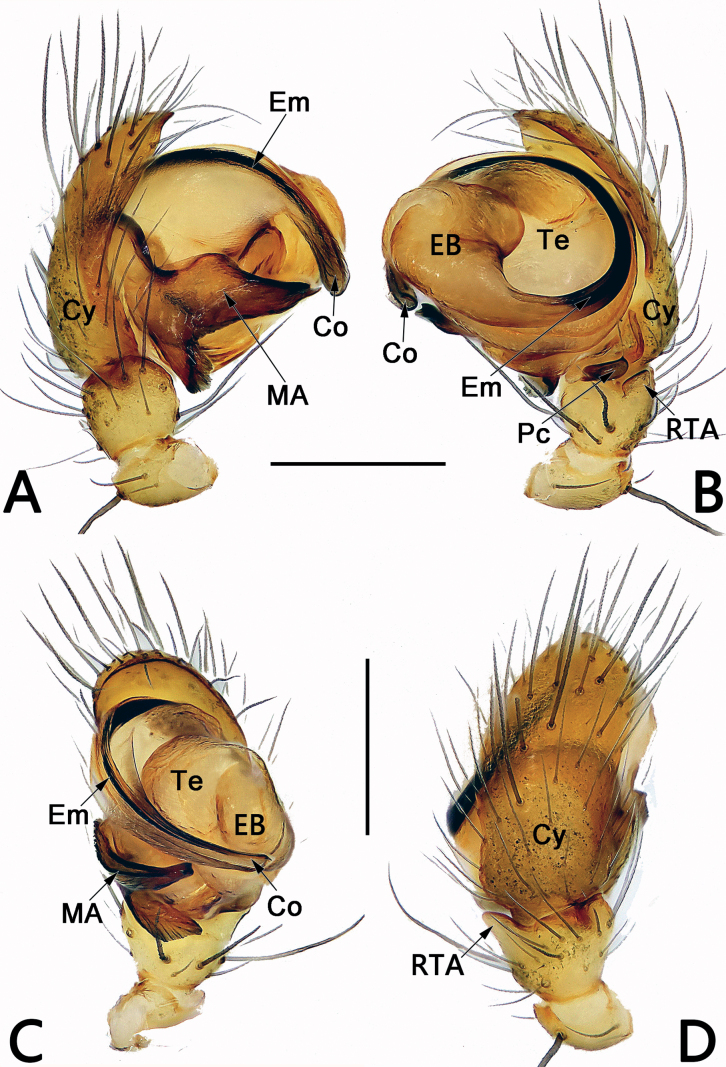
Male palp of the holotype of *Sinoalariachi* sp. nov. **A** prolateral view **B** retrolateral view **C** ventral view **D** dorsal view. Abbreviations: Co = conductor; Cy = cymbium; EB = embolic base; Em = embolus; MA = median apophysis; Pc = paracymbium; RTA = retrolateral tibial apophysis; Te = tegulum. Scale bars: 0.20 mm.

**Figure 8.**
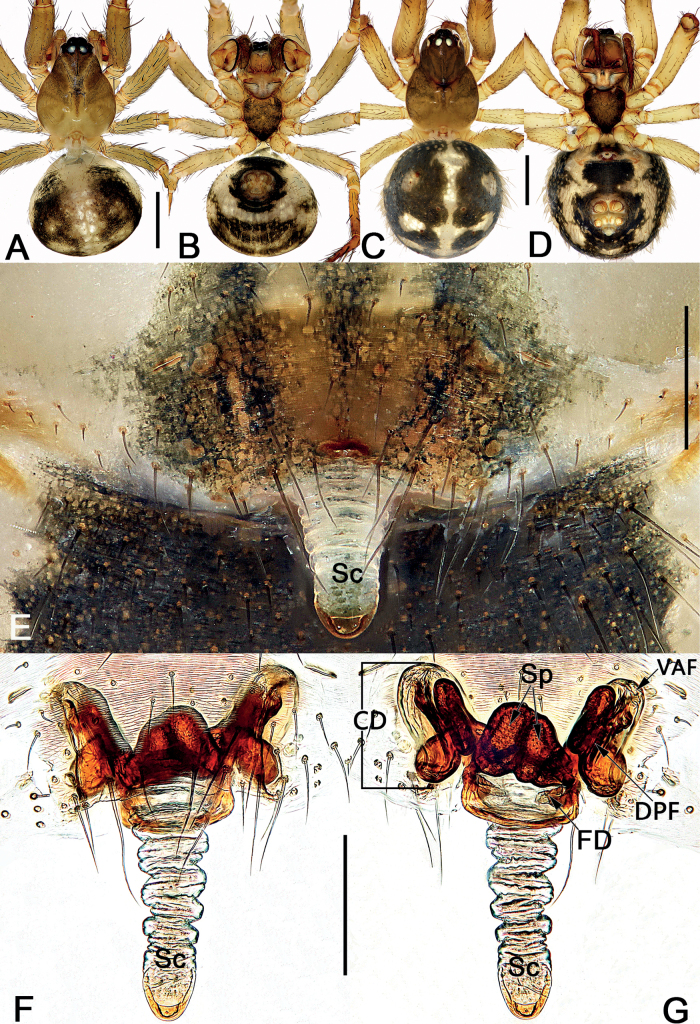
*Sinoalariachi* sp. nov., female paratype and male holotype, male habitus (**A, B**), female habitus (**C, D**) and epigyne (**E–G**) **A** dorsal view **B** ventral view **C** dorsal view **D** ventral view **E** intact, ventral view **F** cleared, ventral view **G** cleared, dorsal view. Abbreviations: CD = copulatory duct; DPF = dorsal and posterior fold of copulatory duct; FD = fertilization duct; Sc = scape; Sp = spermatheca; VAF = ventral and anterior fold of copulatory duct. Scale bars: 0.50 mm (**A–D**); 0.20 mm (**E–G**).

**Figure 9.**
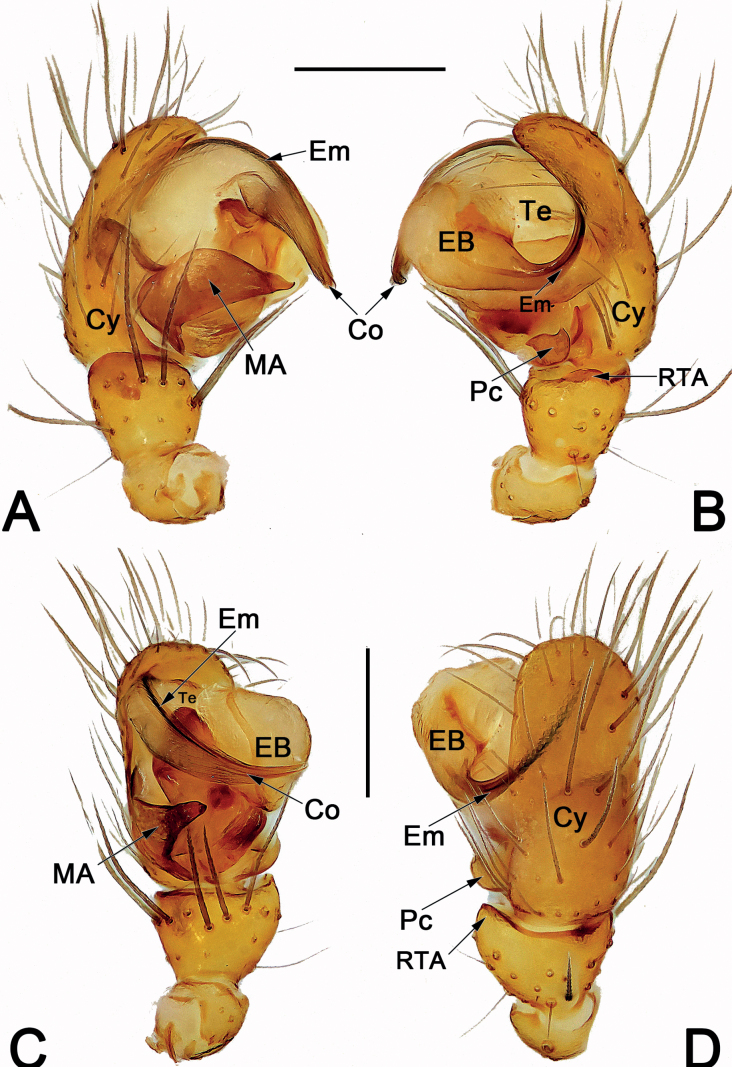
Male palp of *Sinoalarianavicularis* (Lin, Li & Jäger, 2014) **A** prolateral view **B** retrolateral view **C** ventral view **D** dorsal view. Abbreviations: Co = conductor; Cy = cymbium; EB = embolic base; Em = embolus; MA = median apophysis; Pc = paracymbium; RTA = retrolateral tibial apophysis; Te = tegulum. Scale bars: 0.20 mm.

**Figure 10.**
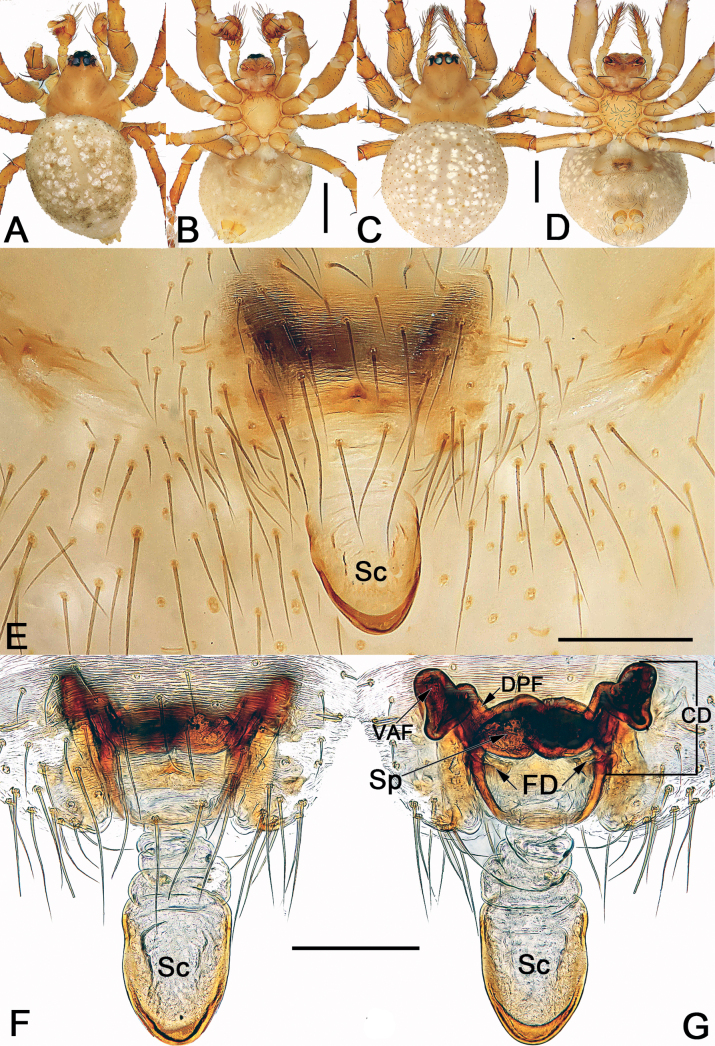
*Sinoalarianavicularis* (Lin, Li & Jäger, 2014), male habitus (**A, B**), female habitus (**C, D**) and epigyne (**E–G**) **A** dorsal view **B** ventral view **C** dorsal view **D** ventral view **E** intact, ventral view **F** cleared, ventral view **G** cleared, dorsal view. Abbreviations: CD = copulatory duct; DPF = dorsal and posterior fold of copulatory duct; FD = fertilization duct; Sc = scape; Sp = spermatheca; VAF = ventral and anterior fold of copulatory duct. Scale bars: 0.50 mm (**A–D**); 0.20 mm (**E–G**).

**Figure 11.**
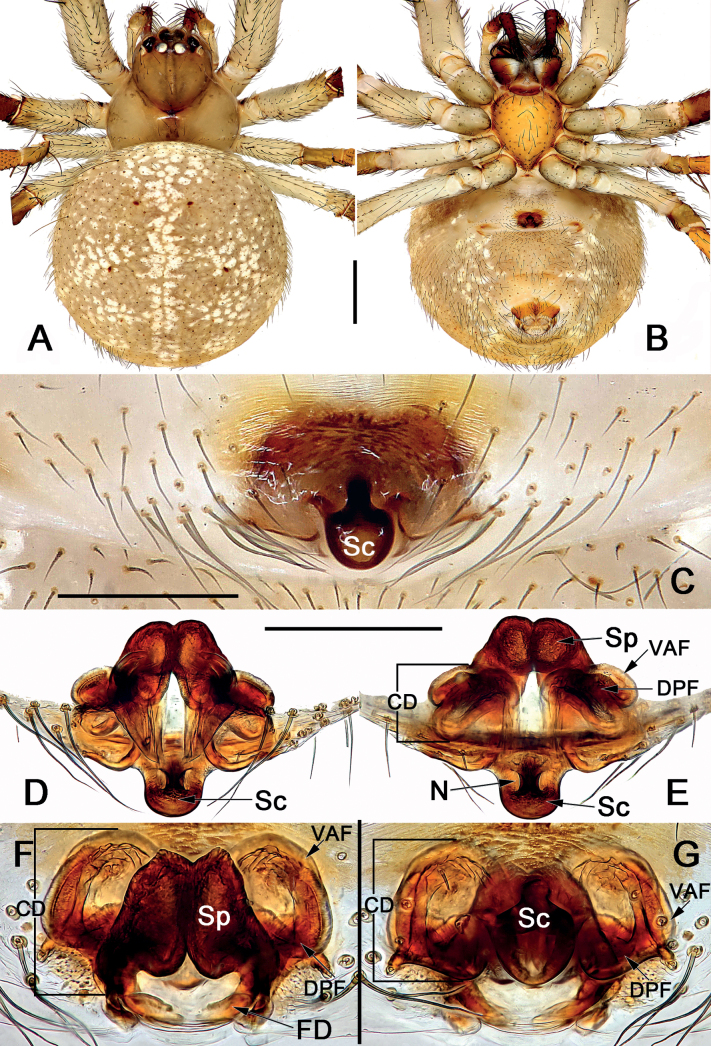
*Sinoalarianitida* (Zhao & Li, 2012), comb. nov., female habitus (**A, B**) and epigyne (**C–G**) **A** dorsal view **B** ventral view **C** intact, ventral view **D** cleared, ventral view **E** cleared, dorsal view **F** anterior view **G** posterior view. Abbreviations: CD = copulatory duct; DPF = dorsal and posterior fold of copulatory duct; FD = fertilization duct; N = notch; Sc = scape; Sp = spermatheca; VAF = ventral and anterior fold of copulatory duct. Scale bars: 0.50 mm (**A, B**); 0.20 mm (**C–G**).

**Figure 12.**
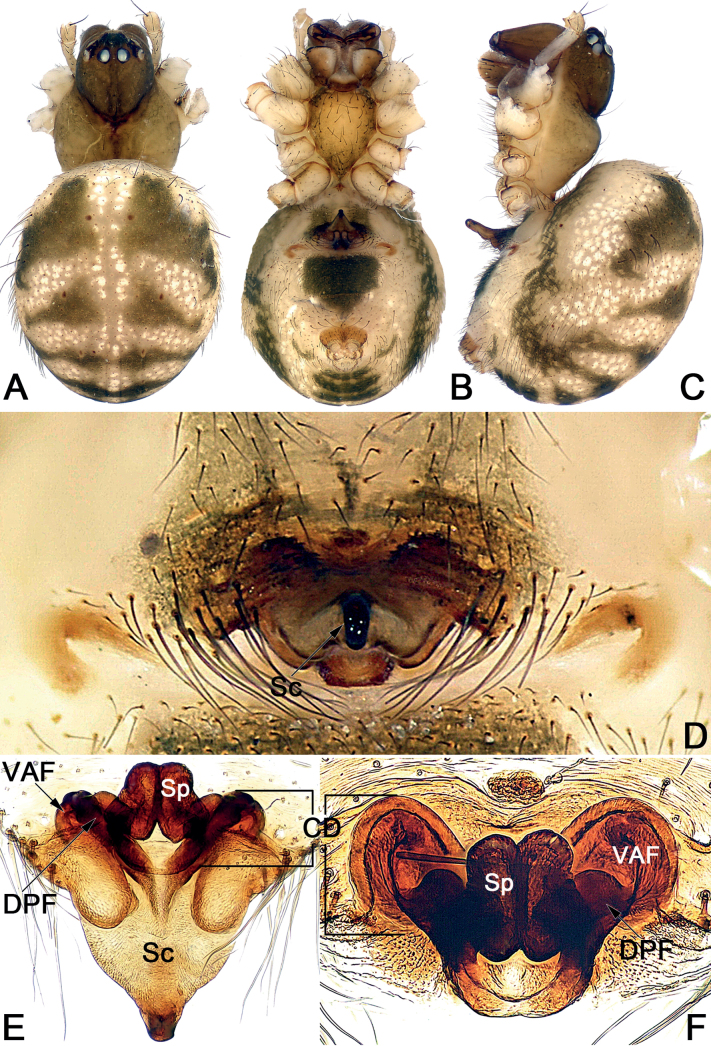
*Sinoalariaprolata* (Zhao & Li, 2012), comb. nov., female habitus (**A–C**) and epigyne (**D–F**) **A** dorsal view **B** ventral view **C** lateral view **D** intact, ventral view **E** cleared, posterior view **F** cleared, anterior view. Abbreviations: CD = copulatory duct; DPF = dorsal and posterior fold of copulatory duct; Sc = scape; Sp = spermatheca; VAF = ventral and anterior fold of copulatory duct. Scale bars: 0.50 mm (**A–C**); 0.20 mm (**D–F**).

**Figure 13.**
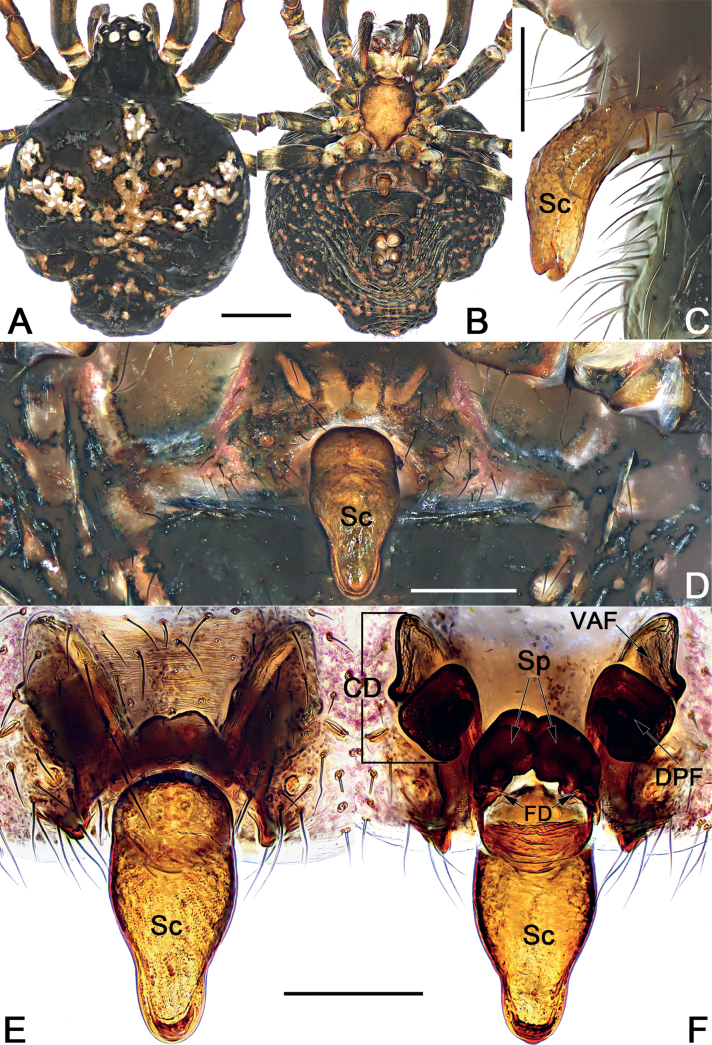
Holotype female of *Sinoalariashenhei* sp. nov., habitus (**A, B**) and epigyne (**C–F) A** dorsal view **B** ventral view **C** intact, lateral view **D** intact, ventral view **E** cleared, ventral view **F** cleared, dorsal view. Abbreviations: CD = copulatory duct; DPF = dorsal and posterior fold of copulatory duct; FD = fertilization duct; Sc = scape; Sp = spermatheca; VAF = ventral and anterior fold of copulatory duct. Scale bars: 0.50 mm (**A, B**); 0.20 mm (**C–F**).

**Figure 14.**
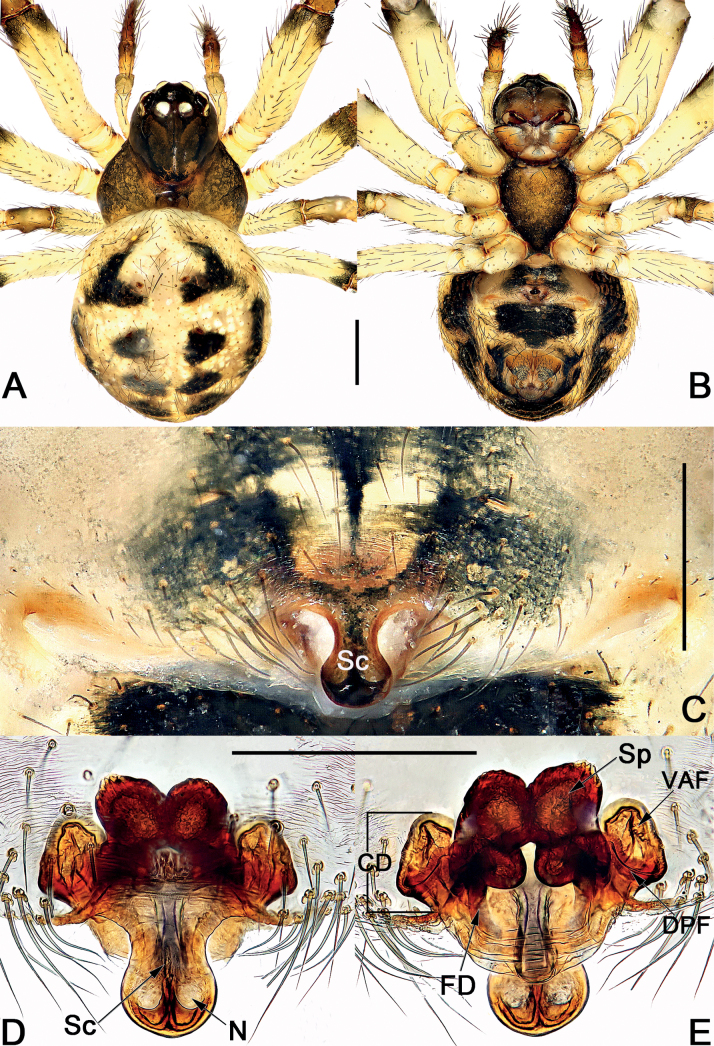
Holotype female of *Sinoalariashuidi* sp. nov., habitus (**A, B**) and epigyne (**C–E) A** dorsal view **B** ventral view **C** intact, ventral view **D** cleared, ventral view **E** cleared, dorsal view. Abbreviations: CD = copulatory duct; DPF = dorsal and posterior fold of copulatory duct; FD = fertilization duct; N = notch; Sc = scape; Sp = spermatheca; VAF = ventral and anterior fold of copulatory duct. Scale bars: 0.50 mm (**A, B**); 0.20 mm (**C–E**).

**Figure 15.**
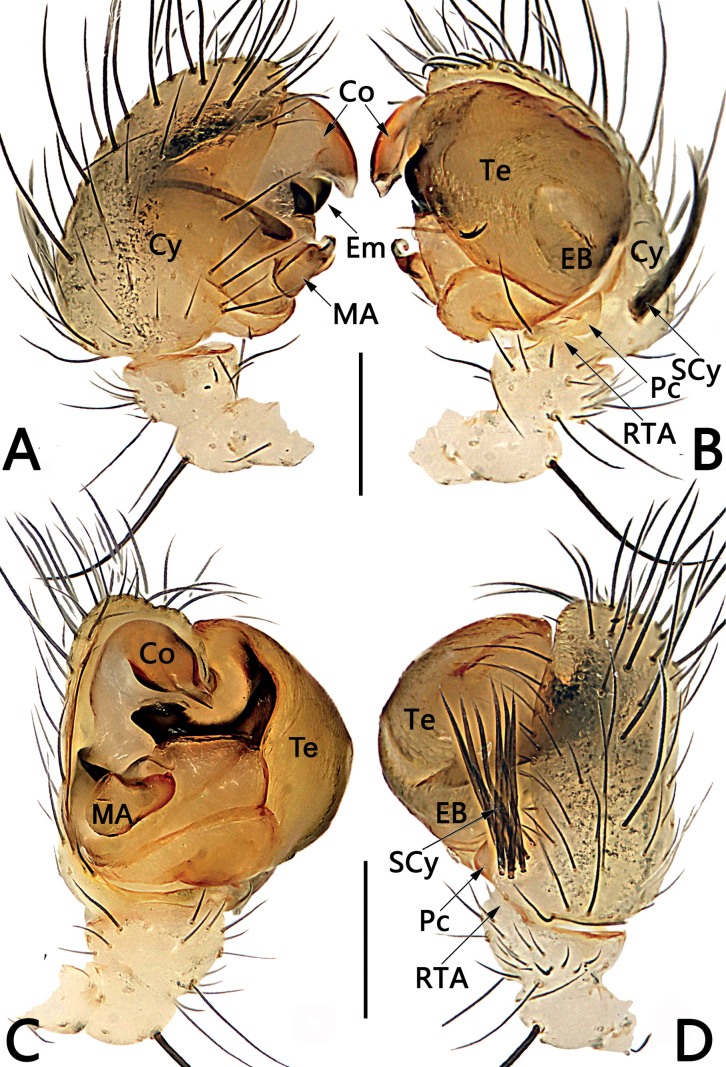
Male palp of the holotype of *Sinoalariaxiaotu* sp. nov. **A** prolateral view **B** retrolateral view **C** ventral view **D** dorsal view. Abbreviations: Co = conductor; Cy = cymbium; EB = embolic base; Em = embolus; MA = median apophysis; Pc = paracymbium; RTA = retrolateral tibial apophysis; SCy = setae on cymbium; Te = tegulum. Scale bars: 0.20 mm.

**Figure 16.**
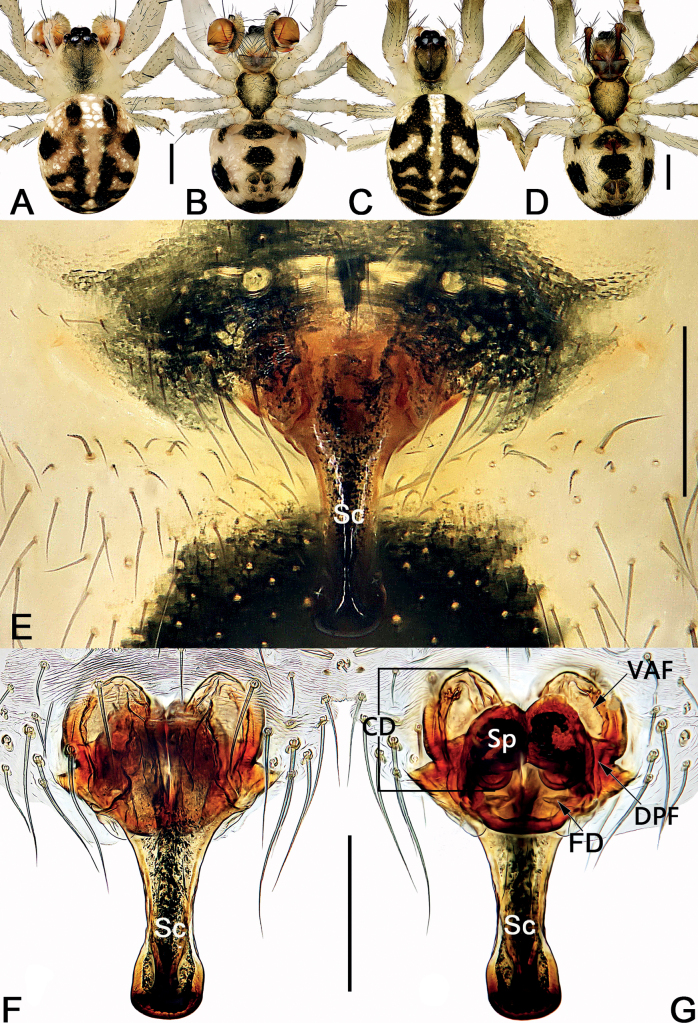
*Sinoalariaxiaotu* sp. nov., female paratype and male holotype, male habitus (**A, B**), female habitus (**C, D**) and epigyne (**E–G**) **A** dorsal view **B** ventral view **C** dorsal view **D** ventral view **E** intact, ventral view **F** cleared, ventral view **G** cleared, dorsal view. Abbreviations: CD = copulatory duct; DPF = dorsal and posterior fold of copulatory duct; FD = fertilization duct; Sc = scape; Sp = spermatheca; VAF = ventral and anterior fold of copulatory duct. Scale bars: 0.50 mm (**A–D**); 0.20 mm (**E–G**).

*Sinoalaria* species can be recognised by the following combination of genitalic characters: In males, palpal tibia retrolaterally bears an apophysis (Figs 1B, D, 3B, 5B, D, 7B, D, 9B, D, 15B, D) (vs retrolateral tibial apophysis is lacking in all other theridiosomatid genera); median apophysis distinct, stretching along the longitudinal axis of pedipalp with two curved, pointed distal ends (Figs 1A, 3A, C, 5A, D, 7A, C, 9A, C, 15A, C) (vs never seen in any other theridiosomatid genus); embolus long and whip-shaped, mostly enveloped in conductor (Figs 1A, C, 3A, C, 5A, C, D, 7A, C, 9A, C, 15A, C) (vs embolus not whip-shaped in almost all of theridiosomatid genera, embolus whip-shaped but proportionately much shorter and partly enveloped in *Ogulnius* O. Pickard-Cambridge, 1882, as in Coddington 1986: figs 100, 101, 116, 118). In females, the epigynal plate possesses a distinct scape (Figs 2E–G, 4E–G, 6D–F, 8E–G, 10E–G, 11C–E, G, 12D, E, 13D–F, 14C–E, 16E–G) (vs scape is absent, or present but reduced in some theridiosomatid genera); vulva centrally with a U-shaped medial structure (Figs 2G, 4G, 6F, 8G, 10G, 11E–G, 12E, F, 13F, 14E, 16G) (vs medial structure lacking, or present but V-shaped in some theridiosomatid genera); copulatory ducts rise and curl up to form two folds (or chambers, or bursae) at each side: the ventral one usually located anteriorly, with lower degree of sclerotization than the dorsal and posterior one (Figs 2G, 4G, 6F, 8G, 10G, 11E–G, 12E, F, 13F, 14E, 16G) (vs such conformation of the copulatory ducts is never seen in any other theridiosomatid genus).

#### Description.

See Zhao and Li (2012).

#### Composition and distribution.

Ten species from southwestern China to Laos, Vietnam and to Thailand: *Sinoalariabicornis* (♂♀) and *S.navicularis* (♂♀) from Laos, *S.chengguanensis* (♂♀), *S.nitida* comb. nov. (♂♀), *S.prolata* comb. nov. (♂♀), *S.shenhei* sp. nov. (♀) and *S.shuidi* sp. nov. (♀) endemic to China, *S.xiaotu* sp. nov. (♂♀) endemic to Vietnam, *S.cavernicola* from Laos and Thailand, *S.chi* sp. nov. (♂♀) from Vietnam and Thailand.

### ﻿Key to species of *Sinoalaria* Zhao & Li, 2014

**Table:** 

1	Males	**2**
–	Females	**7**
2	Cymbium dorsal-basally bears a cluster of several long setae (Figs 1B, D, 5B, D, 15B, D)	**3**
–	Cymbium dorsal-basally without a cluster of several long setae (Figs 3B, D, 7B, D, 9B, D)	**5**
3	Embolic bases prominently visible, median apophysis large, > 1/2 of tegulum length (Fig. 5)	***S.chengguanensis***
–	Embolic base indistinct, median apophysis relatively small, ≤ 1/2 of tegulum length (Figs 1, 15)	**4**
4	Cymbium basally with a cluster of 6 setae; median apophysis extremely small, ≤ 1/3 of tegulum length, both proximal process and distal process are indistinct; the apex of conductor needle-shaped, sharp (Fig. 15)	***S.xiaotu* sp. nov.**
–	Cymbium basally with a cluster of 8 setae; median apophysis comparably larger, ca 1/2 of tegulum length, both proximal process and distal process are distinct; the apex of conductor nearly triangular, relatively blunt (Fig. 1)	***S.bicornis***
5	Median apophysis navicular, proximal process sharp and not serrated, distal process not furcated (Fig. 9A)	***S.navicularis***
–	Median apophysis not navicular, proximal process with a blunt and serrated tip, apex of distal process slightly furcated (Figs 3A, 7A)	**6**
6	Distal process of median apophysis short and wide, slightly shorter and narrower than base of median apophysis; the lower ramus on distal process of median apophysis tooth-shaped, apex sharp, distinctly longer than the indistinct upper ramus; embolus distinctly long, terminating at ca 8 o’clock position in retrolateral view, terminating at ca 4 o’clock position in ventral view; embolic base relatively smaller, its width ca 1/2 of tegulum length (Fig. 7A–C)	***S.chi* sp. nov.**
–	Distal process of median apophysis long and narrow, slightly longer and distinctly narrower than base of median apophysis; both rami on distal process of median apophysis distinct, are of equal length, the lower one with a relatively blunt tip; embolus relatively short, terminating at ca 10 o’clock position both in retrolateral and ventral view; embolic base relatively larger, wider than 2/3 of tegulum length (Fig. 3A–C)	***S.cavernicola***
7	Scape shorter than length of epigynal plate (from anterior level of vulva to posterior margin of epigynal plate) (Figs 11C–E, 14C–E)	**8**
–	Scape longer than length of epigynal plate (Figs 2E–G, 4E–G, 6D–F, 8E–G, 10E–G, 12E, 13D–F, 16E–G)	**9**
8	Scape shaped like a water drop, apex distinctly wider than its stem; spermathecae globular (Fig. 14C–E)	***S.shuidi* sp. nov.**
–	Scape shaped like a nose, apex nearly as wide as stem; spermathecae peanut-shaped (Fig. 11C–E)	***S.nitida***
9	Scape rugose, membranous except slightly sclerotized apex (Figs 2E–G, 4E–G, 8E–G, 10E–G)	**10**
–	Scape not rugose, heavily sclerotized (Figs 6D–F, 12D–F, 13D–F, 16E–G)	**13**
10	Scape wide, ca 1/2 width of epigynal plate, apex nearly triangular (Fig. 10E–G)	***S.navicularis***
–	Scape narrow, ca 1/4–1/3 width of epigynal plate, apex nearly digitiform (Figs 2E–G, 4E–G, 8E–G)	**11**
11	Scape apically with 2 notches, ventral and anterior folds of copulatory ducts slightly sclerotized (Fig. 2F, G)	***S.bicornis***
–	Scape apically with a hood, ventral and anterior folds of copulatory ducts completely membranous (Figs 4E–G, 8E–G)	**12**
12	The ventral and anterior folds of copulatory ducts represented by 2 oblong bursae, the dorsal and posterior folds represented by a longitudinal loop and a horizontal loop (Fig. 8G)	***S.chi* sp. nov.**
–	The ventral and anterior folds represented by 2 globular bursae, the dorsal and posterior folds running horizontally, forming only 1 loop (Fig. 4G)	***S.cavernicola***
13	Scape wide, triangular, protrudes vertically from the posterior epigynal margin; copulatory ducts located laterally to spermathecae (Fig. 12C–F)	***S.prolata***
–	Scape not as above; copulatory ducts located anterolaterally to spermathecae (Figs 6D–F, 13D–F, 16E–G)	**14**
14	Scape shaped like a dumbbell, apically swollen (Fig. 16E–G)	***S.xiaotu* sp. nov.**
–	Scape tongue-shaped or like an inverted bowling pin, apically narrowed (Figs 6D–F, 13D–F)	**15**
15	Scape shaped like an inverted bowling pin, slightly narrowed proximally; the dorsal and posterior folds of copulatory ducts trapeziform, heavily sclerotized; dorsum of abdomen basically black, with three bands which consisting of small white spots, forming a trident-shaped pattern (Fig. 13A, D–F)	***S.shenhei* sp. nov.**
–	Scape tongue-shaped, proximally part distinctly narrowed; the dorsal and posterior folds of copulatory ducts nearly circular, slightly sclerotized; abdomen dorsally white with numerous small black spots (Fig. 6B–F)	***S.chengguanensis***

### ﻿ Sinoalaria bicornis

Taxon classificationAnimaliaAraneaeTheridiosomatidae

(Lin, Li & Jäger, 2014)

EE4F7181-75DF-5F44-B2D8-552D764AEB20

Figs 1 , 2 , 17

Alaria bicornis Lin, Li & Jäger, 2014: 90, figs 11A–D, 12A–F, 13A–E, 14A–C, 15A–F, 16A–C (♂♀).
Sinoalaria bicornis : Zhao and Li 2014: 41.

#### Material examined.

2♂ 6♀, **Laos**: Vien Tiane Province, Vang Vieng District: 13.2 km north of Vieng keo Village, Tham Hoy, 19°02.352'N, 102°25.422'E, 256 m, 3.XII.2012, Z. Yao and S. Li leg.; 4♂ 12♀, 11.95 km north of Vieng keo Village, Pha Thao Cave, 19°01.752'N, 102°25.956'E, 290 m, 3.XII.2012, Z. Yao and S. Li leg.; 3♂ 8♀, 10.37 km north of Vieng keo Village, Kieo Cave, 19°00.882'N, 102°25.902'E, 286 m, 2.XII.2012, Z. Yao and S. Li leg.; 1♂ 7♀, 4.01 km north of Vieng keo Village, Lom Cave, 18°57.456'N, 102°26.244'E, 314 m, 2.XII.2012, Z. Yao and S. Li leg.

#### Diagnosis.

Males of *S.bicornis* resemble those of *S.xiaotu* sp. nov. in the general shape of the male palp. The palps of the two species share the similarly short median apophysis which is ≤ 1/2 of tegulum length, and the indistinct embolic base (Figs 1A, B, 15A–C) (median apophysis relatively large, > 1/2 of tegulum, embolic base prominently visible in all other congeners, including *S.chengguanensis* and *S.chi* sp. nov., etc.; Figs 5, 7), but differ in the following: (1) cymbium basally with a cluster of eight setae in *S.bicornis* (six setae in *S.xiaotu* sp. nov.) (cf. Fig. 1B and Fig. 15B); (2) median apophysis relatively larger, ca 1/2 of tegulum length in *S.bicornis* (extremely small, ≤ 1/3 of tegulum length in *S.xiaotu* sp. nov.) (cf. Fig. 1A, C, D and Fig. 15A, C); (3) conductor with a nearly triangular, relatively blunt apex in *S.bicornis* (with a needle-shaped, sharper apex in *S.xiaotu* sp. nov.) (cf. Fig. 1A, C, D and Fig. 15 A–C). Females resemble those of *S.cavernicola* and *S.chi* sp. nov. in having a distinctly long, narrow, completely membranous, and rugose scape (scape either relatively short and wide, or heavily sclerotized, not rugose in other *Sinoalaria* species, such as *S.shuidi* sp. nov. and *S.xiaotu* sp. nov.; Figs 14C, D, E, 16E, F), but can be distinguished from *S.cavernicola* and *S.chi* sp. nov. by the scape apically with two notch (only with a hood in *S.cavernicola* and *S.chi* sp. nov.) (cf. Fig. 2E, F and Figs 4E, F, 8E, F), ventral and anterior folds of copulatory ducts slightly sclerotized (completely membranous in *S.cavernicola* and *S.chi* sp. nov.) (cf. Fig. 2G and Figs 4G, 8G).

#### Description.

See Lin et al. (2014). Male palp as in Fig. 1, epigyne as in Fig. 2E–G, and habitus as in Fig. 2A–D.

#### Distribution.

Laos (Fig. 17).

### ﻿ Sinoalaria cavernicola

Taxon classificationAnimaliaAraneaeTheridiosomatidae

(Lin, Li & Jäger, 2014)

D47A080E-A3B4-54EA-9FFE-5DC9FC8B5F4B

Figs 3 , 4 , 17

Alaria cavernicola Lin, Li & Jäger, 2014: 77, figs 1A–H, 2A–E, 3A–G, 4A, B (♂♀).
Sinoalaria cavernicola : Zhao and Li 2014: 41.

#### Material examined.

1♂ 10♀, **Laos**: Ban Kouanphavang Khammouane Province: 30.02 km northeast of Thakhek Town, Tham Kamouk Cave, 17°37.914'N, 104°07.458'E, 193 m, 24.XI.2012, Z. Yao and S. Li leg.; 2♂ 5♀, **Thailand**: Satun Province: Thung wa District, Cave without name-cave A & B, 07°03.966'N, 99°50.478'E, 12 m, 1–3.XII.2013, F. Ballarin leg.; 1♂ 8♀, Thung wa District, Cave without name,07°06.276'N, 99°47.502'E, 25 m, 29.XI.2013, F. Ballarin leg.

#### Diagnosis.

Males of *S.cavernicola* and *S.chi* sp. nov. share the following features: the proximal process of median apophysis with a blunt and serrated tip, and the distal process of median apophysis with a slightly furcated apex (Figs 3A, 7A) (proximal process not serrated, distal process not furcated in all other *Sinoalaria* species, including *S.chengguanensis* and *S.navicularis*; Figs 5A, 9A), but can be differ from the latter by: (1) distal process of median apophysis long and narrow, slightly longer and distinctly narrower than base of median apophysis in *S.cavernicola* (short and wide, slightly shorter and narrower than base of median apophysis in *S.chi* sp. nov.) (cf. Fig. 3A and Fig. 7A); (2) both rami on distal process of median apophysis distinct, are of equal length, the lower one with a relatively blunt tip in *S.cavernicola* (the lower ramus tooth-shaped, apex sharp, distinctly longer than the indistinct upper ramus in *S.chi* sp. nov.) (cf. Fig. 3A and Fig. 7A); (3) embolic base relatively larger, wider than 2/3 of tegulum length in *S.cavernicola* (relatively smaller, its width ca 1/2 of tegulum length in *S.chi* sp. nov.) (cf. Fig. 3B and Fig. 7B); (4) embolus relatively shorter, terminating at ca 10 o’clock position both in retrolateral and ventral view in *S.cavernicola* (distinctly longer, terminating at ca 8 o’clock position in retrolateral view, terminating at ca 4 o’clock position in ventral view in *S.chi* sp. nov.) (cf. Fig. 3B, C and Fig. 7B, C). Females of *S.cavernicola* are also similar to those of *S.chi* sp. nov. by the epigynal plate with a long, completely membranous, rugose scape which distally with a pocket-like hood, and by the similar configurations of vulva (Figs 4E–G, 8E–G), but they can be differentiated by the shapes and the courses of copulatory ducts: the ventral and anterior folds represented by two nearly globular bursae, the dorsal and posterior folds running horizontally, forming only one loop in *S.cavernicola* (Fig. 4G); in contrast, the ventral and anterior folds represented by two oblong bursae, the dorsal and posterior folds represented by a longitudinal loop and a horizontal loop in *S.chi* sp. nov. (Fig. 8G).

#### Description.

See Lin et al. (2014). Male palp as in Fig. 3, epigyne as in Fig. 4E–G, and habitus as in Fig. 4A–D.

#### Distribution.

**Figure 17.**
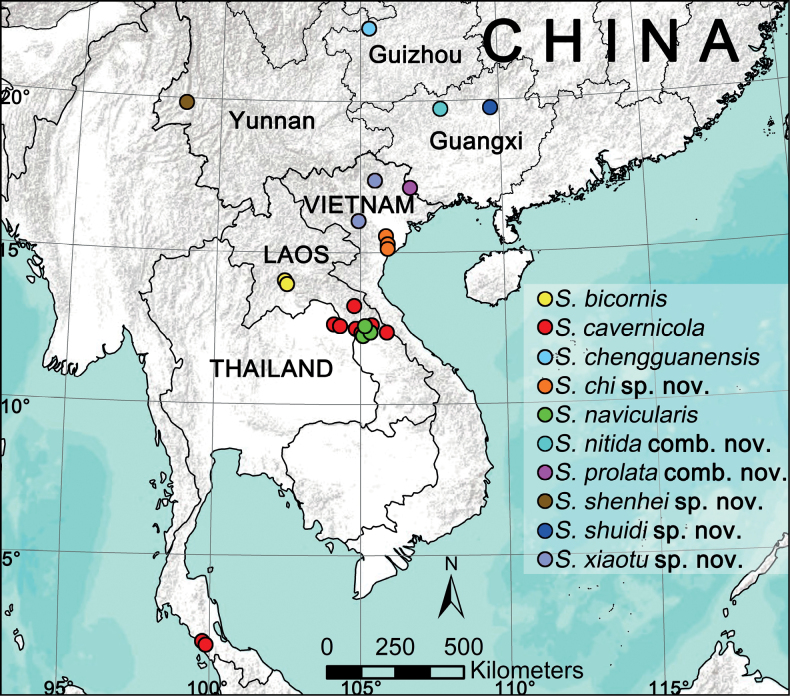
Distribution records of the species from genus *Sinoalaria*.

Laos and Thailand (Fig. 17).

### ﻿ Sinoalaria chengguanensis

Taxon classificationAnimaliaAraneaeTheridiosomatidae

(Zhao & Li, 2012)

97AB83E3-DDBC-5ECD-AFBC-17CF38C09EE1

Figs 5 , 6 , 17

Alaria chengguanensis Zhao & Li, 2012: 8, figs 1A–D, 2A–D, 3A–D, 4A–F, 5A–D (♂♀).
Sinoalaria chengguanensis : Zhao and Li 2014: 41.

#### Material examined.

***Holotype*** ♂ and ***paratypes*** 13♂ **8**♀ **(IZCAS)**, **China**: Guizhou Province, Bijie City, Chengguan Town, Xiaohe Village, Xiniu Cave, 27°21.231'N, 105°17.186'E, 1515 m, 30.IV.2007, J. Liu and Y. Lin leg.

#### Diagnosis.

Males of *S.chengguanensis* resembles those of *S.bicornis* and *S.xiaotu* sp. nov. by their cymbium dorsal-basally bears a cluster of several long setae (Figs 1B, D, 5B, D; 15B, D) (such cluster of several long setae is absent in all other *Sinoalaria* species, as in Figs 3, 7, 9), but can be distinguished from the latter two by the distinctly visible embolic base, and by the large median apophysis which is longer than 1/2 of tegulum length in *S.chengguanensis* (embolic base indistinct, median apophysis relatively small, no more than 1/2 of tegulum length in *S.bicornis* and *S.xiaotu* sp. nov.) (cf. Fig. 5 and Figs 1, 15). The female of *S.chengguanensis* appears to be closely related to *S.navicularis* and *S.shenhei* sp. nov. in having a similarly shield-shaped, utterly exposed scape, and similar configurations of vulva (Figs 6D–F, 10D–F, 13D–F), but can be recognised from *S.navicularis* by the scape heavily sclerotized, with a small transverse opening at its distal end (vs almost completely membranous, rugose, distally with a curved, pocket-like hood) (cf. Fig. 6D–F and Fig. 10D–F); and from *S.shenhei* sp. nov. can be recognised by the tongue-shaped scape with a distinctly narrowed proximal part (vs scape shaped like an inverted bowling pin, slightly narrowed proximally) (cf. Fig. 6D–F and Fig. 13D–F), the dorsal and posterior folds of copulatory ducts nearly circular, slightly sclerotized (vs trapeziform, heavily sclerotized) (cf. Fig. 6F and Fig. 13F), and by the abdomen dorsally white with numerous small black spots (vs dorsum of abdomen basically black, with three bands which consisting of white small spots, forming a trident-shaped pattern) (cf. Fig. 6B, C and Fig. 13A).

#### Description.

See Zhao and Li (2012). Male palp as in Fig. 5, epigyne as in Fig. 6D–F, and habitus as in Fig. 6A–C.

#### Distribution.

China (Guizhou) (Fig. 17).

### ﻿ Sinoalaria chi

Taxon classificationAnimaliaAraneaeTheridiosomatidae

Yu & Lin sp. nov.

20F70BAC-4029-5063-9E5F-12A8B196661C

https://zoobank.org/1F9C4A04-5DC7-47ED-A404-FADDA21C7946

Figs 7 , 8 , 17

#### Type material.

***Holotype*** ♂ and ***Paratype*** 1♀, **Vietnam**: Hang Dai Ca, 20°33.520'N, 105°53.287'E, 30.XII.2013, H. Sterner leg.

#### Other material examined.

**Vietnam.** 1♂ 2♀, Hang Boi, 20°15.533'N, 105°53.278'E, 24–25.XII.2013, H. Steiner leg.; 1♂ 5♀, Hang Tra Tu, 20°06.492'N, 105°55.040'E, 28.XII.2013, H. Steiner leg.

#### Etymology.

The specific name is derived from the Chinese pinyin ‘chǐ’, which means ‘tooth’, referring to the tooth-shaped lower ramus on distal process of median apophysis; noun in apposition.

#### Diagnosis.

Males of *S.chi* sp. nov. resemble those of *S.cavernicola*. The two species share a similarly slender and filiform embolus, sheet-shaped paracymbium with a spine-like tip, and the median apophysis with serrated proximal process and slightly bifurcated distal process but differ by: (1) distal process of median apophysis short and wide, slightly shorter and narrower than the base of median apophysis in *S.chi* sp. nov. (long and narrow, slightly longer and distinctly narrower than base in *S.cavernicola*) (cf. Fig. 7A and Fig. 3A); (2) the lower ramus on distal process of median apophysis tooth-shaped, apex sharp, distinctly longer than the indistinct upper ramus in *S.chi* sp. nov. (both rami distinct, are of equal length, the lower one with a relatively blunt tip in *S.cavernicola*) (cf. Fig. 7A and Fig. 3A); (3) embolus distinctly longer, terminating at ca 8 o’clock position in retrolateral view, terminating at ca 4 o’clock position in ventral view in *S.chi* sp. nov. (terminating at ca 10 o’clock position both in retrolateral and ventral view in *S.cavernicola*) (cf. Fig. 7A, C and Fig. 3A, C); (4) embolic base relatively smaller, its width ca 1/2 of tegulum length in *S.chi* sp. nov. (embolic base wider than 2/3 of tegulum length in *S.cavernicola*) (cf. Fig. 7B and Fig. 3B). Females also resemble those of *S.cavernicola* in having a completely membranous, rugose scape which distally with a pocket-like hood (the combination of these features are absent in all other congeners), and the general shape of the endogyne but can be distinguished from the latter by the ventral and anterior folds of copulatory ducts represented by two oblong bursae, the dorsal and posterior folds represented by a longitudinal loop and a horizontal loop in *S.chi* sp. nov. (ventral and anterior folds represented by two more or less globular bursae, dorsal and posterior folds running horizontally, forming only one loop in *S.cavernicola*) (cf. Fig. 8G and Fig. 4G).

#### Description.

**Male** (holotype) (Fig. 8A, B): Carapace shaped like a water drop, brown, darker in the front, without distinct pattern; pars cephalica distinctly narrowed, cervical groove and radial grooves faint. Anterior eye row distinctly recurved, posterior eye row slightly procurved. Sternum shield-shaped, dark. Mouthparts reddish brown. Legs uniformly yellowish, femora slightly darker. Abdomen round, dorsum basically black, with a lengthwise yellowish median stripe extending almost of whole abdomen length, gradually narrow posteriorly, with three pairs of yellowish speckles on either side; venter black centrally, yellowish marginally. *Measurements*: Total length 2.4. Carapace 1.1 long, 0.8 wide. Clypeus 0.1 high. Sternum 0.5 long, 0.4 wide. Abdomen 1.1 long, 1.1 wide. Length of legs: I 3.1 (1.1, 0.3, 0.7, 0.6, 0.4); II 2.3 (0.7, 0.3, 0.5, 0.5, 0.3); III 1.7 (0.5, 0.2, 0.5, 0.3, 0.2); IV 2.3 (0.8, 0.3, 0.6, 0.4, 0.2).

***Palp*** (Fig. 7A–D): Tibia small, cup-shaped, ca 1/3 length of cymbium. Retrolateral tibial apophysis small but distinct, thumb-like, ca 1/3–1/2 length of tibia. Cymbium narrow, ca 2.3× longer than wide, dorsally bears several long and sparse setae, basally without tufty setae. Paracymbium ca 1/8–1/7 length of cymbium, represented by a small sheet, distally bears a needle-shaped spine. Tegulum capacious, oval, 1.3× longer than wide; sperm duct distinct. Median apophysis large, located prolaterally to tegulum, consisting of a broad base and two process; proximal process ca 2/3 length of base, distally serrated; distal process wide, slightly shorter than base, distally slightly bifurcated, upper ramus indistinct, lower ramus tooth-shaped. Embolic base large, represented by enlarged tubercle, located at the 9–10 o’clock position to tegulum in retrolateral view, its width ca 1/2 tegulum length. The free part of embolus long and slender, filiform, in retrolateral view, arising at approximately the 8–9 o’clock position, forming a loop, terminating at ca 8 o’clock position, its tip curved behind embolic base. Conductor tubular and translucent, enveloping the second half of embolus, apex pointing retrolaterally.

**Female** (paratype). Somatic features as in Fig. 8C, D and coloration distinctly darker than in male. *Measurements*: Total length 3.2. Carapace 1.5 long, 1.1 wide. Clypeus 0.1 high. Sternum 1.1 long, 0.9 wide. Abdomen 2.1 long, 1.6 wide. Length of legs: I 4.4 (1.4, 0.4, 1.1, 1.0, 0.5); II 3.5 (1.1, 0.4, 0.9, 0.7, 0.4); III 2.4 (0.6, 0.3, 0.6, 0.5, 0.3); IV 3.2 (1.0, 0.3, 0.8, 0.7, 0.4).

***Epigyne*** (Fig. 8C–E). Epigynal plate large, distinctly wider than long, the arrangement of the various parts of the vulva are faint through the tegument. Scape long, rugose, translucent, extending from posterior margin of epigynal plate, ca 1.8× plate length; apex blunt and slightly sclerotized, represented by a small pocket-like hood. Copulatory ducts strongly convoluted, located laterally to spermathecae: ventral and anterior folds of copulatory ducts represented by two oblong bursae, bursae surface membranous, ca 2/3 length of epigyne plate, the two bursae separated by ca 1.7× their widths; dorsal posterior folds tubular, heavily sclerotized and strongly convoluted, forming a longitudinal loop and a horizontal loop, finally connecting with ventral surface of spermathecae. Spermathecae bean-shaped, strongly sclerotized, ca 1/2 length of epigyne plate, 2× longer than wide, located centrally and juxtaposed, the two spermathecae closely spaced. Fertilization ducts short, ribbon-shaped, membranous, located on dorsal-basal surface of spermathecae; apical parts separated by ca 1.5× fertilization duct length, apex curved and sharp.

#### Distribution.

Northern Vietnam (Fig. 17).

### ﻿ Sinoalaria navicularis

Taxon classificationAnimaliaAraneaeTheridiosomatidae

(Lin, Li & Jäger, 2014)

B59E7D59-395C-5014-BA81-5D32E50934CB

Figs 9 , 10 , 17

Alaria navicularis Lin, Li & Jäger, 2014: 83, figs 5A–F, 6A–D, 7A–D, 8A–E, 9A–D, 10A–C (♂♀).
Sinoalaria navicularis : Zhao and Li 2014: 41.

#### Material examined.

1♂ 4♀, **Laos**: Khammouan Province, 2.5 km WNW, Ban Tathot, Eingang, 17°37.897'N, 103°07.502'E, 200 m, 20.II.2003, P. Jäger leg.; 4♀, Tham Koun Dôn, 17°333.82'N, 104°52.132'E, 24.II.2006, H. Steiner leg.

#### Diagnosis.

Males of *S.navicularis* can be distinguished from all other congeners by the navicular median apophysis (Fig. 9A). The female of *S.navicularis* is distinguished from other congeners except *S.chengguanensis* and *S.shenhei* sp. nov. by the scape with a moderate size, utterly exposed, like a shield attached to the abdomen (Figs 6D, 10E, 13D) (scape not as above, either distintly long and narrow, such as *S.bicornis*, *S.cavernicola*, *S.chi* sp. nov. and *S.xiaotu* sp. nov. as in Figs 2E–G, 4E–G, 8E–G, 16E–G, or distintly short and wide, such as *S.nitida* and *S.shuidi* sp. nov. as in Figs 11C–E, 14C–E), but differ from the latter two by the scape almost completely membranous, rugose (more sclerotized, not rugose in *S.chengguanensis* and *S.shenhei* sp. nov.) (cf. Fig. 10E–G and Figs 6D–F, 13D–F).

#### Description.

See Lin et al. (2014). Male palp as in Fig. 9, epigyne as in Fig. 10 E–G, habitus as in Fig. 10A–D.

#### Distribution.

Laos (Fig. 17).

### ﻿ Sinoalaria nitida

Taxon classificationAnimaliaAraneaeTheridiosomatidae

(Zhao & Li, 2012) comb. nov.

6162C5AB-DC8E-5E6C-9947-A5C9634217E7

Figs 11 , 17

Karstia nitida Zhao & Li, 2012: 20, figs 11A–E, 12A, B (♀).

#### Material examined.

***Paratype*** 1♀ **(IZCAS)**, **China**: Guangxi: Hechi City, Jinchengjiang District, Hechi Town, Laba Village, Shoushui Cave, 24°41.229'N, 107°52.609'E, 268 m, 31.III.2011, Z. Chen and Z. Zha leg. Examined.

#### Diagnosis.

*Sinoalarianitida* morphologically is similar to *S.shuidi* sp. nov. by the scape shorter than length of epigynal plate, with a swollen apex (Figs 11C–E, 14C–E) (scape longer than length of epigynal plate, distally not swollen in all other congeners, such as *S.chengguanensis*, *S.chi* sp. nov., *S.shenhei* sp. nov. and *S.xiaotu* sp. nov.; Figs 6E, F, 8E, F, 13E, F, 16E, F). From *S.shuidi* sp. nov., *S.nitida* can be distinguished by the following features: (1) the scape shaped like a nose, apex nearly as wide as stem (shaped like a water drop, apex distinctly wider than its stem in *S.shuidi* sp. nov.) (cf. Fig. 11C–E and Fig. 14C, D); (2) the spermathecae peanut-shaped (globular in *S.shuidi* sp. nov.) (cf. Fig. 11E, F and Fig. 14E).

#### Description.

See Zhao and Li (2012). Habitus as in Fig. 11A, B, epigyne as in Fig. 11C–G.

#### Distribution.

China (Guangxi) (Fig. 17).

### ﻿ Sinoalaria prolata

Taxon classificationAnimaliaAraneaeTheridiosomatidae

(Zhao & Li, 2012) comb. nov.

67F89FBF-D1ED-516A-82F3-25185DDC5FF5

Figs 12 , 17

Karstia prolata Zhao & Li, 2012: 23, figs 13A–F, 14A, B (♀).

#### Material examined.

***Holotype*** ♀ (IZCAS), **China**: Guangxi, Pingxiang City, Youyi County, Bantou Village, Niuyan Cave, 22°05.666'N, 106°45.439'E, 251 m, 18.I.2011, Z. Chen and Z. Zha leg. Examined.

#### Diagnosis.

This species can be distinguished from all other species of the genus *Sinoalaria* by the wide, triangular, scape that protrudes vertically from the posterior epigynal margin (Fig. 12C–E).

#### Description.

See Zhao and Li (2012). Habitus as in Fig. 12A–C, epigyne as in Fig. 12D–F.

#### Distribution.

China (Guangxi) (Fig. 17).

### ﻿ Sinoalaria shenhei

Taxon classificationAnimaliaAraneaeTheridiosomatidae

Yu & Lin sp. nov.

8BDEC8BB-4CA0-5996-9CA6-A88C134B5B12

https://zoobank.org/3D838714-E6C5-4405-B200-3FFF4DE34C00

Figs 13 , 17

#### Type material.

***Holotype*** ♀, **China**: Yunnan, Baoshan City, Longling County, Longjiang Town, Xiaoheishan Provincal Nature Reserve, 24°49.733'N, 98°45.601'E, 2020 m, 22.VIII.2018, Y. Lin et al. leg.

#### Other material examined.

11 juv., same data as holotype.

#### Etymology.

The specific name is an adjective and derived from the Chinese pinyin ‘shēn hēi’, which means ‘pitch-black’, referring to the basic color of body.

#### Diagnosis.

The new species is similar to *S.chengguanensis* (Fig. 6) in the general appearance of the epigyne. From *S.chengguanensis*, the female of *S.shenhei* sp. nov. can be distinguished by the shape of the scape, the different shape and degrees of sclerotization of copulatory ducts, as well as the color of habitus: (1) scape shaped like an inverted bowling pin, slightly narrowed proximally in *S.shenhei* sp. nov. (scape tongue-shaped, proximal part distinctly narrowed in *S.chengguanensis*) (cf. Fig. 13D–F and Fig. 6D–F); (2) dorsal and posterior folds of copulatory ducts trapeziform, heavily sclerotized in *S.shenhei* sp. nov. (nearly circular, slightly sclerotized in *S.chengguanensis*) (cf. Fig. 13F and Fig. 6F); (3) dorsum of abdomen basically black, with three bands which consisting of small white spots, forming a trident-shaped pattern in *S.shenhei* sp. nov. (abdomen dorsally white with numerous small black spots in *S.chengguanensis*) (cf. Fig. 13A and Fig. 6B, C).

#### Description.

**Female** (holotype) (Fig. 13A, B): Carapace nearly pyriform, uniformly black; cervical groove and radial grooves faint. Anterior eye row recurved, posterior eye row almost straight in dorsal view. Sternum shield-shaped, centrally light orange with sparse setae, marginally dark. Mouthparts yellowish. Legs dark brown except black femur. Abdomen nearly round, posteriorly with a prominent caudo-dorsal hump, covered with sparse long setae, setal base sclerotized. Dorsum of abdomen basically black, with three bands consisting of small white spots forming a trident-shaped pattern. Venter of abdomen black, centrally with numerous brown small spots. *Measurements*: Total length 3.5. Carapace 1.3 long, 1.1 wide. Clypeus 0.1 high. Sternum 0.7 long, 0.6 wide. Abdomen 2.5 long, 2.4 wide. Length of legs: I 3.4 (1.1 0.3, 0.9, 0.7, 0.4); II 2.5 (0.6, 0.3, 0.6, 0.6, 0.4); III 2.2 (0.5, 0.3, 0.5, 0.6, 0.3); IV 3.0 (1.0, 0.3, 0.5, 0.6, 0.3).

***Epigyne*** (Fig. 13C–F). Epigynal plate nearly as wide as long, spermathecae and copulatory ducts are faint through epigynal plate before dissection. Scape as long as epigynal plate, shaped like an inverted bowling pin, apex with a pocket-like hood; protruding from concaved posterior margin of epigynal plate. Copulatory ducts coils located anterolaterally to spermathecae: ventral and anterior folds represented by two hyaline and triangular bursae, ca 1/3 length of epigyne plate, the two folds widely separated by ca 2.1× their width; the dorsal and posterior fold trapezoid, heavily sclerotized, separated by ca 1.3× their diameters. Spermathecae fist-shaped, strongly sclerotized, located centrally and juxtaposed, not overlapping with copulatory ducts; the two spermathecae touch each other. Fertilization ducts short, acicular, membranous, located on posterior surface of spermathecae.

**Male.** Unknown.

#### Distribution.

Known only from the type locality (Fig. 17).

### ﻿ Sinoalaria shuidi

Taxon classificationAnimaliaAraneaeTheridiosomatidae

Yu & Lin sp. nov.

C89A44F6-E549-5D30-9D62-D4246112EFB8

https://zoobank.org/E1C0B653-BF85-47FC-8BA5-938B019D0A6E

Figs 14 , 17

#### Type material.

***Holotype*** ♀ and ***Paratypes*** 3♀, **China**: Guangxi, Liuzhou City, Luzhai County, Zhongdu Town, Xiamotun Village, Jiulong Cave, 24°44.917'N, 109°39.844'E, 257 m, 15.VII.2013, H. Zhao leg.

#### Etymology.

The specific name is derived from the Chinese pinyin ‘shuǐ dī’, which means water drop, referring to the scape that is shaped like a water drop; noun in apposition.

#### Diagnosis.

This new species is similar to *S.nitida* in having the scape shorter than length of epigynal plate, with a swollen apex (Figs 11C–E, 14C–E) (scapes of all other congeners, such as *S.chengguanensis*, *S.chi* sp. nov., *S.shenhei* sp. nov. and *S.xiaotu* sp. nov., which are no shorter than the length of epigynal plate, distally not swollen; Figs 6E, F, 8E, F, 13E, F, 16E, F), but can be distinguished by the scape shaped like a water drop, apex distinctly wider than its stem in *S.shuidi* sp. nov. (scape shaped like a nose, apex nearly as wide as stem in *S.nitida*), and by the spermathecae globular in *S.shuidi* sp. nov. (peanut-shaped in *S.nitida*) (cf. Fig. 14C–E and Fig. 11C–G).

#### Description.

**Female** (holotype) (Fig. 14A, B): Carapace pear-shaped, pars cephalica distinctly dark in ocular area; cephalic region distinctly narrowed, cervical groove distinctly delimited, radial grooves distinguishable and vein-shaped. Anterior eye row recurved, posterior eye row almost straight in dorsal view. Sternum shield-shaped, centrally dark yellowish brown, marginally dark, with sparse setae. Mouthparts yellowish brown. Legs yellow, all legs with conspicuous dark annuli in the distal parts of femur, and patella. Abdomen spherical, covered with sparse long setae, setal base sclerotized; dorsum basically yellow, with four pair of black spots: 1^st^ pair <-shaped, the other three pairs nearly triangular; venter yellow, centrally with a cup-shaped black speckle. *Measurements*: total length 3.0. Carapace 1.4 long, 1.1 wide. Clypeus 0.2 high. Sternum 0.7 long, 0.6 wide. Abdomen 2.0 long, 1.5 wide. Length of legs: I 5.7 (1.7, 0.6, 1.3, 1.3, 0.8); II 4.7 (1.5, 0.6, 0.9, 0.9, 0.8); III 3.3 (0.8, 0.5, 0.7, 0.8, 0.5); IV 4.3 (1.5, 0.5, 0.9, 0.9, 0.5).

***Epigyne*** (Fig. 14C–E). Epigynal plate nearly as wide as long, spermathecae and copulatory ducts are faint through epigynal plate before dissection. Scape relatively short, ca 3/4 of length of epigynal plate, with a slightly narrow stem and swollen apex, shaped like a water drop, apex with two pocket-like notches; proximal part originating from posterior portion of the plate. Copulatory ducts coils located laterally to spermathecae: ventral and anterior folds represented by two oval bursae, bursae surface hyaline, ca 1/2 length of epigyne plate, the two bursae separated by ca 2× their widths; dorsal and posterior folds heavily sclerotized, tubular, thick, not convoluted, extending horizontally to connect with ventral surface of spermathecae. Spermathecae nearly globular, strongly sclerotized, located centrally and juxtaposed, overlapping with distal part of dorsal and posterior folds of copulatory ducts; spermathecal diameter ca 2/5 length of epigyne plate, the two spermathecae closely spaced. Fertilization ducts short, acicular, membranous, located on posterior surface of spermathecae; apical parts separated by ca 2× fertilization duct, apex sharp.

**Male.** Unknown.

#### Distribution.

Known only from the type locality (Fig. 17).

### ﻿ Sinoalaria xiaotu

Taxon classificationAnimaliaAraneaeTheridiosomatidae

Yu & Lin sp. nov.

D882587D-4FC2-5728-91C2-87D3AC7B54FE

https://zoobank.org/52B075C8-D0FB-4D35-9692-E067165CF667

Figs 15 , 16 , 17

#### Type material.

***Holotype*** ♂ and ***Paratypes*** 6♀, **Vietnam**: Bac Can, Barbie National Park, Beilan cave, 22°22.766'N, 105°36.790'E, 280 m, 2.IV.2012, Z. Yao leg.; 1♂ 6♀, same data as holotype, 18.X.2012, H. Zhao and Z. Chen leg.

#### Other material examined.

1♂ 2♀, **Vietnam**: Phu Tho, Xuan Son National Park, Lun Cave, 21°07.022'N, 104°57.443'E, 398 m, 26.X.2012, H. Zhao and Z. Chen leg.

#### Etymology.

The specific name is derived from the Chinese pinyin ‘xiǎo tū’, which means ‘small apophysis’, referring to the small median apophysis which is no more than 1/3 of tegulum length; noun in apposition.

#### Diagnosis.

Males of *S.xiaotu* sp. nov. can be distinguished from congeners except *S.bicornis* by the similar indistinct embolic base and the small median apophysis which is no more than 1/2 of tegulum length (Figs 1A, B, 15A–C) (embolic bases prominently visible, large median apophysis longer than 1/2 of tegulum length in all other *Sinoalaria* species, including *S.chengguanensis* and *S.chi* sp. nov., etc.; Figs 5, 7), but differ from the latter by the: (1) median apophysis extremely small, no more than 1/3 of tegulum length, both proximal process and distal process are indistinct in *S.xiaotu* sp. nov. (median apophysis relatively larger, ca 1/2 of tegulum length, proximal process finger-like, distal process hook-shaped in *S.bicornis*) (cf. Fig. 15A–C and Fig. 1A); (2) cymbium basally with a cluster of six setae in *S.xiaotu* sp. nov. (with a cluster of eight setae in *S.bicornis*) (cf. Fig. 15D and Fig. 1B); (3) the apex of conductor needle-shaped, sharp in *S.xiaotu* sp. nov. (nearly triangular, relatively blunt in *S.bicornis*) (cf. Fig. 15C and Fig. 1A). Females of this species can be distinguished from all other congeners by the heavily sclerotized and dumbbell-shaped scape (Fig. 16E, F).

#### Description.

**Male** (holotype) (Fig. 16A, B): Carapace nearly pyriform, yellowish white, with a wide U-shaped paramedian stripe starting from behind PLE, almost reaching the posterior margin of carapace; paramedian stripe centrally dark, marginally lighter. Anterior eye row distinctly recurved, posterior eye row almost straight in dorsal view. Sternum heart-shaped, centrally yellowish with sparse setae, marginally dark. Mouthparts yellowish brown. Legs uniformly yellowish white. Abdomen oval, clothed with dense setae. Dorsum of abdomen basically yellowish brown, with four pair of black spots: 1^st^ pair oval and separated by ca 1.5× width; 2^nd^ pair nearly fan-shaped, widely separated by ca 3× width; 3^rd^ pair and 4^th^ pair fused, represented by a pattern which is shaped like ‘][’. Venter of abdomen yellowish white, centrally with a pentagon-shaped black speckle. *Measurements*: Total length 1.8. Carapace 1.0 long, 0.8 wide. Clypeus 0.1 high. Sternum 0.5 long, 0.4 wide. Abdomen 1.2 long, 1.1 wide. Length of legs: I 3.6 (1.2, 0.4, 1.0, 0.6, 0.4); II 3.2 (1.0, 0.4, 0.9, 0.6, 0.3); III 2.3 (0.6, 0.3, 0.6, 0.5, 0.3); IV 2.8 (0.7, 0.3, 0.7, 0.7, 0.4).

***Palp*** (Fig. 15A–D): Tibia small, cup-shaped, ca 1/5–1/4 length of cymbium, with several short and sparse setae. Retrolateral tibial apophysis small, ca 1/4–1/3 length of tibia, nearly triangular, apex blunt. Cymbium narrow, ca 2.2× longer than wide, dorsally bears several long and sparse setae; basally with a cluster of six setae, the setae conspicuous and almost 1/2 of cymbial length. Paracymbium blade-shaped, ca 1/6–1/5 length of cymbium, apex acute. Tegulum broad, oval, 1.4× longer than wide, central surface with reticular grooves, marginally smooth; sperm duct distinct. Median apophysis small, ≤ 1/3 of tegulum length, with a papilliform proximal process and a digitiform distal process, both processes are indistinct. Embolus filiform, in retrolateral view, arising at approximately the 4–5 o’clock position, extending behind tegulum, terminating at ca 10 o’clock position, embolic base indistinct. Conductor tubular and relatively sclerotized, thick except needle-shaped apex.

**Female** (one paratype). Somatic features as in Fig. 16C, D and coloration slightly darker than in male. *Measurements*: Total length 2.2. Carapace 1.0 long, 0.7 wide. Clypeus 0.1 high. Sternum 0.5 long, 0.4 wide. Abdomen 1.4 long, 1.1 wide. Length of legs: I 3.4 (1.0, 0.3, 0.8, 0.9, 0.4); II 2.4 (0.7, 0.3, 0.5, 0.6, 0.3); III 1.9 (0.5, 0.2, 0.4, 0.5, 0.3); IV 2.7 (0.9, 0.3, 0.6, 0.6, 0.3).

***Epigyne*** (Fig. 16E–G). Epigynal plate slightly wider than long, the arrangement of the various parts of the vulva obscured through epigynal plate before dissection. Scape long, heavily sclerotized, surface smooth; proximal part fuse to ventral plate of epigyne, originating from posterior portion of the plate, extending posteriorly, ca 1.3× length of the plate; proximally widest, then gradually narrowing distally, finally widening at apex, shaped like a dumbbell-shaped. Copulatory ducts coils located anterolaterally to spermathecae: ventral and anterior folds represented by two large bursae, bursae surface hyaline and egg-shaped; almost all part of dorsal and posterior folds covered by posterior part of spermathecae. Spermathecae nearly globular, strongly sclerotized, located centrally and juxtaposed, overlapping with dorsally postero-interior part of copulatory ducts; spermathecal diameter ca 1/3 length of epigyne plate, the two spermathecae closely spaced. Fertilization ducts short, acicular, membranous, located on posterior surface of spermathecae; apical parts separated by approximately the length of fertilization duct, apex sharp.

#### Distribution.

Known only from Barbie National Park and Xuan Song National Park in Vietnam (Fig. 17).

## ﻿Discussion

The genus *Sinoalaria* shows a distinct set of genital characters, such as palpal tibia retrolaterally bearing an apophysis in male, and copulatory ducts rising and curling up to form two folds (or chambers, or bursae) at each side in females. In contrast, the RTA is lacking and such conformation of the copulatory ducts has never been seen in any other theridiosomatid genus; therefore, *Sinoalaria* can be separated from all other genera of Theridiosomatidae. Furthermore, a preliminary molecular analysis of Theridiosomatidae from Southeast Asia was carried out, based on five targeted genes (two mitochondrial genes 16S and COI; three nuclear genes 18S, 28S, and H3). According to the unpublished results, the monophyly of *Sinoalaria* is strongly supported.

*Sinoalarianitida* and *S.prolata* were assigned to the genus *Karstia* in the original publication (Zhao and Li 2012), although they show typical *Sinoalaria* features: copulatory ducts rise and curl up to form two folds (or chambers, or bursae) in both species (Figs 11E–G, 12E–F). The females of *Karstia* species (known for *K.upperyangtzica* Chen, 2010 and *K.cordata* Dou & Lin, 2012 and several unpublished new species) share the following distinctive suite of characters, here contrasted with the corresponding condition in *S.nitida* and *S.prolata*:

1. scape large and semi-transparent, shaped nearly like an equilateral triangle, with an acute apex and two straight lateral margins, protruding posteriorly (Chen 2010: 3, fig. 5; Zhang and Wang 2017: 826, 6 figs; Dou and Lin 2012: 734, figs 16–18) (vs scape short in *S.nitida*, long but shaped like an acute triangle in *S.prolata*, and in both species, scape are more sclerotized, and more or less protruding perpendicularly, apex blunt, two lateral margins not straight; Figs 11C–E, 12D, E);
2. the two spermathecae nearly arranged horizontally, only overlapped each other at the tip (Chen 2010: 3, fig. 6; Zhang and Wang 2017: 826, 6 figs; Dou and Lin 2012: 734, fig. 18) (vs spermathecae nearly arranged longitudinally, located centrally and juxtaposed, nearly touched each other along the inner face; Figs 11E, F, 12E, F);
3. copulatory ducts short, not curled (Chen 2010: 3, fig. 6; Zhang and Wang 2017: 826, 6 figs; Dou and Lin 2012: 734, fig. 18) (vs copulatory ducts curling up to form two folds; Figs 11E–G, 12E, F).

In view of the above-mentioned facts, it is currently impossible to discern any obvious derived features that could indicate a close relationship between the two species and the genus *Karstia*, leaving no doubts that our transfer is correct.

## Supplementary Material

XML Treatment for Sinoalaria

XML Treatment for Sinoalaria bicornis

XML Treatment for Sinoalaria cavernicola

XML Treatment for Sinoalaria chengguanensis

XML Treatment for Sinoalaria chi

XML Treatment for Sinoalaria navicularis

XML Treatment for Sinoalaria nitida

XML Treatment for Sinoalaria prolata

XML Treatment for Sinoalaria shenhei

XML Treatment for Sinoalaria shuidi

XML Treatment for Sinoalaria xiaotu

## ﻿Additional information

### Author ORCIDs

Jianshuang Zhang https://orcid.org/0000-0003-4010-3082

Hao Yu https://orcid.org/0000-0002-9113-2425

Yucheng Lin https://orcid.org/0000-0002-5054-0633

### Data availability

All of the data that support the findings of this study are available in the main text.
